# Comparing sexual risks and patterns of alcohol and drug use between injection drug users (IDUs) and non-IDUs who report sexual partnerships with IDUs in St. Petersburg, Russia

**DOI:** 10.1186/1471-2458-10-676

**Published:** 2010-11-05

**Authors:** Nadia Abdala, Edward White, Olga V Toussova, Tatiana V Krasnoselskikh, Sergei Verevochkin, Andrei P Kozlov, Robert Heimer

**Affiliations:** 1 Department of Epidemiology and Public Health, Yale University School of Medicine, New Haven, CT, USA; 2 The Biomedical Center, St. Petersburg, Russian Federation

## Abstract

**Background:**

To date, the great majority of Russian HIV infections have been diagnosed among IDUs and concerns about the potential for a sexual transmission of HIV beyond the IDU population have increased. This study investigated differences in the prevalence of sexual risk behaviors between IDUs and non-IDUs in St. Petersburg, Russia and assessed associations between substance use patterns and sexual risks within and between those two groups.

**Methods:**

Cross-sectional survey data and biological test results from 331 IDUs and 65 non-IDUs who have IDU sex partners were analyzed. Multivariate regression was employed to calculate measures of associations.

**Results:**

IDUs were less likely than non-IDUs to report multiple sexual partners and unprotected sex with casual partners. The quantity, frequency and intensity of alcohol use did not differ between IDUs and non-IDUs, but non-IDUs were more likely to engage in alcohol use categorized as risky per the alcohol use disorders identification test (AUDIT-C). Risky sexual practices were independently associated with monthly methamphetamine injection among IDUs and with risky alcohol use among non-IDUs. Having sex when high on alcohol or drugs was associated with unprotected sex only among IDUs.

**Conclusions:**

Greater prevalence of sexual risk among non-IDUs who have IDU sex partners compared to IDUs suggests the potential for sexual transmission of HIV from the high-prevalence IDU population into the general population. HIV prevention programs among IDUs in St. Petersburg owe special attention to risky alcohol use among non-IDUs who have IDU sex partners and the propensity of IDUs to have sex when high on alcohol or drugs and forgo condoms.

## Background

To date the great majority of Russian HIV infections and AIDS cases have been diagnosed among injection drug users (IDUs) {Population Reference Bureau, 2007 #574; Shaboltas, 2006 #384; Kozlov, 2006 #385} and previous research indicates that drug injection is markedly more prevalent than a decade ago [4]. In Russia and elsewhere in the former Soviet states, the drugs most commonly injected are heroin and ephedrine derivates, including methamphetamine [5-7]. Both categories of drugs have been independently associated with sexual practices that risk HIV transmission or acquisition [6,8,9]. In addition, alcohol consumption in Russia has substantially increased in the turbulent years following the collapse of the Soviet Union [10,11] and is reportedly among the highest in the world [12]. Heavy alcohol consumption, which is prevalent among IDUs in Russia, has been associated with sexual risk-taking and injection practices that facilitate HIV transmission [13,14].

Heavy use of illicit injectable drugs and alcohol has led to concerns about the potential for a sexual transmission of HIV into the general population. There are several factors that determine whether HIV transmission will be limited to the core population of injectors in which it is prevalent or if it will become a generalized epidemic [15]. Among these are the size and activity levels of the bridging population, which is comprised of those who form partnerships with both IDUs and with non-IDUs, and partnership formation patterns within the general population [16,17]. Studies conducted among Russian non-drug using youth indicate that in recent years levels of unprotected sex have increased, age of sexual debut has decreased, rates of partner change have increased, and sexual activity under the influence of alcohol has become more prevalent among the general population, increasing the potential for the transmission of sexually transmitted infections (STIs) overall [18-20]. While HIV transmission-related behavior is prevalent among Russian IDUs [21-23], it is not known whether such behavior is more or less prevalent among their non-IDU partners. In addition, while a number of substance-use-related patterns have been associated with sexual risk among Russian IDUs, association between sexual risk and substance use has not been estimated for non-IDUs who have IDU sex partners.

The current analysis was conducted using data from a study in St. Petersburg that was part of a large multi-site project, the Sexual Acquisition and Transmission of HIV  Cooperative Agreement Program (SATH-CAP) [24]. The goal of SATH-CAP was to collect and analyze data on sexual and drug use practices and other social, environmental and biological factors that may influence HIV transmission, from high-prevalence core populations, i.e. IDUs and men who have sex with men (MSM) to the greater general populations of which they are part. This analysis compared the sexual risk behaviors and patterns of substance use between heterosexual IDUs and non-IDUs who have IDU sexual partners with the following aims: (1) to investigate whether substance use measures in both groups differed in their correlations with sexual risks, and (2) to determine whether sexual risk behaviors are associated with global substance use levels and with substance use in sexual contexts.

## Methods

### Study Population and Recruitment into the SATH-CAP study

The SATH-CAP study protocol was approved by the Institutional Review Boards of Yale University, the Biomedical Center in St. Petersburg, and RAND Corporation. Participants were recruited between November 2005 and December 2008 using a modified form of respondent driven sampling (RDS) [24,25]. RDS is a chain-referral sampling methodology that uses structured incentives and coupon disbursement procedures for peer referrals. Conventional RDS methods were modified [24] in this study to recruit both core members (IDUs and MSM) and their sex partners whether they were core members or not. Briefly, enrolled core members were given coupons to distribute to their core peers and to their sex partners. Potential participants after they received coupons had to call to schedule an appointment and to show up at the study site for eligibility screening and enrollment. Newly enrolled cores were in turn offered coupons to recruit additional core peers and their own sexual partners. Participation was confidential. No locator information was collected but a number of bio-measures including forearm length and wrist circumference were collected to prevent repeat participation (duplicates).

Participants completed structured interviews and were given pre-test counseling before biological specimens were collected for the testing of sexually transmitted infections, including HIV. Participants received incentives including mobile phone cards and personal care items; all received subway tokens, condoms and HIV prevention information. Participants were instructed when to return to the site to receive their laboratory test results and post-test counseling. Participants were referred for other medical services as needed.

### Interview Data Collection

Interviews lasting 90-120 minutes were conducted using computer-assisted survey interviewing technology on laptop computers. Information was collected on socio-demographics, health status and HIV-associated sexual and injection risk behaviors. Socio-demographic items included age, sex, marital status, education, employment and source of income, housing and whether the participant considers him or herself homeless or had ever been jailed. Self-reported health data included HIV testing and results, illicit injection drug use (IDU) in the last 6 months, the number of times injected in the last month, whether the participant used methamphetamines -including amphetamines or ephedrine-in the past month, whether the participant used syringes or injection equipment that had been used by others for injection. Participants were asked three items from the Alcohol Use Disorders Identification Test (AUDIT-C) instrument: frequency of alcohol use in the last month, the usual number of alcoholic drinks consumed at each drinking event, and frequency in which participant got drunk or consumed five or more drinks in two hours in the last month. Sexual behaviors in the six months prior to interview included number of male and female sex partners, number of sex partnerships of less than three months duration, sex partnership type (i.e. main or non-main), vaginal and anal sex with and without condoms at the last sexual encounter with up to five of their most recent sexual partners, and for each reported partnership, whether the participant or his or her partner had been "high" on drugs or alcohol during one of their sexual encounters.

### STI Detection Assays

Blood and urine specimens were obtained from all participants. Serum was tested for human immunodeficiency virus type-1 (HIV-1) and syphilis. HIV-1 testing involved two HIV-1 antibodies enzyme immunological assays (EIAs) (Vironostika HIV-1, Uni-Form-II plus-0; Biomerieux, Netherlands and Genscreeen HIV-1/2; BioRad, France). Positive HIV-1 EIA tests were confirmed by Western Blot (WB) (HIV-1 WB Type-1, Biorad, France). Serum samples were screened for syphilis with rapid plasma reagin test (RPR) (Macro-Vue RPR-Card Tests, Becton Dickinson, USA) and *Treponema pallidum *particle agglutination assay (TPPA) (Serodia-TPPA, Fujirebio, Japan). Specimens were seropositive for syphilis if results of both tests were reactive. Urine specimens were tested for nucleic acid from *Chlamydia trachomatis *(chlamydia) and *Neisseriae gonorrheae *(gonorrhea) using polymerase chain reaction (Amplicor CT/NG, Roche, USA).

### Hypotheses Testing and Data Analysis

This study investigated the prevalence of sexual risk behaviors, non-viral STIs and substance use patterns between IDUs and non-IDUs who have IDU sex partners and tested three hypotheses: (1) that the prevalence of STIs and the frequency of sexual risk behaviors would be greater among IDUs than non-IDUs, (2) that substance use measures that were correlated with sexual risks would be different among IDUs and non-IDUs, and (3) that both global patterns of substance use and substance use in sexual contexts would be associated with unprotected sex with casual partners among both IDUs as well as among non-IDUs.

The criteria for inclusion into this analysis were: injection drug use in the last six months or having a sex partner who is an IDU; testing negative for HIV or being unaware of positive HIV status upon enrollment; if male, reporting only sex with females; being sexually active in the last six months; and providing data on sexual partnerships. Because sexual partnership information may have been duplicated when participants recruited their own sexual partners into the study, sexual partnerships reported by recruits with his or her recruiter were excluded from the analysis. Thus participants were excluded from the analysis if their only reported sex partner was his or her recruiter.

Three dimensions of HIV-related sexual risks served as separate outcomes: (1) reporting more than one sex partner in the prior six months, i.e. multiple sex partners, (2) reporting unprotected sex with casual partners, i.e. with non-main or new (< 3 months) sex partners, and (3), testing positive for a non-viral STI (*N. gonorrheae, C. trachomatis *and/or syphilis serology). We chose non-viral STIs as an outcome because these STIs represent recent sexual risk taking [26] and since they are not transmitted through sharing injection equipment they are suitable markers for the comparison of sexual risks between IDUs and non-IDUs.

Risky alcohol use corresponded to AUDIT-C scores greater than three for males and greater than two for females based on answers to the first three AUDIT questions as determined in previous studies [27-29]. Unsafe injection was defined as using syringes or equipment that had been already used by another IDU. Having sex when high on drugs or alcohol referred to participants who reported having sex while he/she or any partner was drunk or "high" on drugs.

All analyses were performed using Stata for Macintosh version 10. Descriptive statistics were used to compare, between IDUs and non-IDUs, the prevalence of demographic, socio-economic measures, sexual behavior and non-viral STIs, alcohol consumption patterns including AUDIT-C scores, and having sex when high on drugs or alcohol. Methamphetamine use was assessed for both IDUs and non-IDUs. For IDUs, descriptive statistics included injection frequency and unsafe injection. Differences between IDUs and non-IDUs were assessed using Chi-square tests, or for non-normally distributed continuous variables, Wilcoxon rank-sum test.

Bivariate and multivariate logistic regressions were conducted for all participants who fit inclusion criteria, and for IDUs and non-IDUs separately. Logistic regression was employed to assess bivariate associations of the three selected sexual risk behavior outcomes (multiple sex partners, unprotected sex, and a positive test for a non-viral STI) with the demographic and substance use related factors. Multivariate logistic regression was conducted to produce unconfounded estimates of associations between each of the above outcomes and demographic and substance-use patterns. Multivariate models were constructed by including all covariates that were statistically significant at alpha level ≤0.2 in the bivariate analysis and gradually removing each non-significant covariate until all covariates were statistically significant (p < 0.05) or removal did not change adjusted odds ratio point estimates by > 10%. Because measures of injection frequency and unsafe injection were relevant for IDUs but not for non-IDUs, associations between those covariates and outcomes were assessed for IDUs but not for non-IDUs. Similarly, injection drug in the prior six months was relevant only in models that included both IDUs and non-IDUs. Thus, multivariate models inclusive of all participants and those stratified by IDU status, had the potential to include overlapping, but slightly different subsets of covariates.

Since participants could report more than one sexual partner, we analyzed the risks factors related to condom use at last intercourse within each sexual partnership using generalized estimating equations (GEE).

Interactions between drug injection in the prior 6 months and three substance use related variables: at-risk AUDIT-C score, methamphetamine use, and having sex when high on drugs or alcohol were investigated. Interaction terms between drug injection and each substance use related variable were created separately, added one by one to the models, and compared with main effect models using likelihood-ratio tests. Risk factors were considered to be significantly different between IDUs and non-IDUs when an interaction term was significant after being added to the main effects model and the likelihood-ratio test confirmed its improvement of the overall fit of the model.

## Results

### General Characteristics of Subjects

In all, 1,023 IDUs, MSM and their sexual partners were recruited into the completed SATH-CAP study in St. Petersburg. After excluding HIV-infected participants who were aware of being HIV positive (146), were MSM (242), reported zero sex partners (57), did not report number of sex partners in the prior six months (93), did not answer questions about sexual partnerships (29) and had as their only sex partner the participant who recruited them into the study (60), 396 participants were included in the analysis. These included 84% (331/396) who reported IDU in the prior six months and 16% (65/396) who did not (Table 1). Among all participants included in this analysis, 68% (269/396) were male; age ranged from 18 to 53 years with a median of 28 years (IQR = 24-32). IDUs were more likely to be older (Table 1), male, married, have uncompleted postsecondary education, be unemployed, live with family, report no legal income, and have been incarcerated.

**Table 1:** Comparing prevalence of substance use and sexual risk practices between heterosexual IDUs (n = 331) and non-IDUs (n = 65)

	IDU	non-IDU	
	n (%)*^1 ^	n (%)*^1 ^	p-value
**Demographics**			
Age (median, IQR)	29 (25-32)	24 (21-32)	< 0.001*
Male sex	243 (73)	26 (40)	< 0.001
Married/partnered	135 (41)	15 (23)	0.009
Postsecondary education	191 (58)	50 (77)	0.004
Employed	147 (44)	51 (80)	< 0.001
Live with family	196 (59)	27 (42)	0.009
Any legal income	250 (76)	58 (89)	0.015
Jail	116 (36)	2 (3)	< 0.001
Homeless	61 (18)	9 (14)	0.376

**Sexual behavior of subjects in the past 6 months**			
Had > 1 sex partner	141 (43)	44 (68)	< 0.001
Any casual partner	145 (44)	43 (66)	0.001
Had a new (< 90 days) partner	172 (58)	38 (62)	0.491
Unprotected sex at last intercourse with any partner	183 (59)	44 (71)	0.074
Unprotected sex at last intercourse with casual partners*^2 ^	114 (34)	37 (57)	0.001
Had sex when high on drugs or alcohol*^3 ^	228 (70)	35 (55)	0.017

**Sexually transmitted infections**			
Tested positive for HIV antibodies	90 (27)	2 (3)	< 0.001
Tested positive for a non-viral STI*^4^	19 (6)	7 (11)	0.166

**Alcohol consumption in the past month**			
Risky alcohol use*^5 ^	208 (66)	50 (85)	0.004
Days used alcohol last 30 days (median, IQR)	5 (2-15)	5 (2-10)	0.816*
Drinks on average each time drinking (median, IQR)	3 (2-5)	3 (2-5)	0.667*
Days had > 4 drinks in 2 hours or got drunk (median, IQR)	1 (0-4)	1 (0-3)	0.687*

Illicit drug consumption in the past month*^6^			
Meth use*^7 ^	60 (18)	7 (11)	0.155

**Injection practices in the last 6 months**			
Unsafe injection, 30 days	82 (25)		
Number of times injected last 30 days, median (p25-75)	20 (8-30)		

* Wilcoxon rank-sum test

*^1 ^among participants who replied the question

*^2 ^i.e. had unprotected sex with non-main or new (< 90 days) sexual partners

*^3 ^had sex while participant or partner or both were drunk or high

*^4 ^*Chlamydia trachomatis, Neisseiae gonorrheae *and/or syphilis serology

*^5 ^determined by an at-risk AUDIT-C score

*^6 ^IDUs injected mostly heroin and meth. All other drugs such as cocaine, crack, speedball were < 7% prevalence among

IDUs as well as among non-IDUs

*^7 ^i.e. amphetamine, ephedrine or methamphetamine. Among IDUs 99% of meth was consumed in the form of injection

### Sexual Behavior and STI Outcomes

In bivariate analysis, non-IDUs were significantly more likely than IDUs to report having had multiple sex partners, casual partners, and unprotected sex at last intercourse with casual partners, i.e. with new (< 30 days) or non-main partners. IDUs were more likely than non-IDUs to report having sex while the participant, his or her partner, or both were drunk or high (i.e. having sex when high on drugs or alcohol) (Table 1).

The prevalence of non-viral STIs was low and did not significantly differ between IDUs and non-IDUs. Among IDUs, the prevalence of chlamydia, gonorrhea and syphilis serology was 4% (14/331), 1% (4/331) and 1% (3/331), respectively. Two participants were co-infected with chlamydia and gonorrhea. Among non-IDUs, the prevalence of chlamydia, gonorrhea and syphilis serology was 9% (6/65), 2% (1/65) and 0%, respectively; there were no co-infections. Non-viral STI prevalence did not differ by gender, but prevalence was too low for the analysis to have any statistical power.

### Substance Use

Among IDUs who reported the types of drugs used in the last month, the drug most frequently used was heroin or opiates at 84% followed by methamphetamine at 18%. Nearly all IDUs (99%) reported having taken opiates and methamphetamine in the form of injection and the use of other types of hard drugs was limited (< 7%). A quarter (82/331) reported using syringes or equipment that had been used by someone else. The median monthly injection frequency was 20 (Table 1). Eleven percent of non-IDUs reported consuming non-injectable methamphetamine.

Non-IDUs were significantly more likely than IDUs to receive at-risk AUDIT-C scores. However, the median number of days they consumed alcohol per month, the median number of drinks at each drinking event, and the median number of days participants "got drunk" or had > 4 drinks in two hours in the previous 30 days did not significantly differ between IDUs and non-IDUs.

### Correlates of Testing Positive for a Non-viral STI

Testing positive for a non-viral STI was independently associated with younger age (aOR = 0.1; 95% C.I. 0.0-0.6; p = 0.016) and with having multiple sex partners (aOR = 2.7; 95% C.I. 1.1-6.4; p = 0.024). Among IDUs, no variable was independently associated with non-viral STI. Insufficient observations did not permit an analysis among non-injectors.

### Correlates of Having Multiple Sexual Partners

In multivariate logistic regressions using all eligible participants reporting multiple partnerships was independently associated with being non-IDU, methamphetamine use, and reporting sex when high on drugs or alcohol (Table 2). In analysis restricted to IDUs, reporting multiple sex partners was independently associated with methamphetamine use and reporting sex when high on drugs or alcohol. Among non-IDUs, having multiple sex partners was independently associated with having sex while high on drugs or alcohol and marginally associated with risky alcohol use. The latter was retained in the multivariate model for non-IDUs due to its removal changing the aOR for sex while high by > 10%.

**Table 2:** Associations between substance use practices and having multiple sex partners for IDUs and non-IDUs

	All participants	N = 396	IDUs	n = 331	Non-IDUS	n = 65
	uOR	aOR*^4 ^	uOR	aOR*^4 ^	uOR	aOR*^4 ^
Male	0.7 (0.5-1.1)*		0.8 (0.5-1.3)		1.1 (0.4-3.3)	
HIV	0.8 (0.5-1.3)		1.0 (0.6-1.6)		0.5 (0.03-7.8)	
Log age	0.9 (0.4-2.2)		0.7 (0.3-2.2)		7.2 (0.8-68.5)*	
Married/partnered	0.8 (0.5-1.2)		0.8 (0.5-1.3)		1.3 (0.4-4.8)	
Injection drug use last 6 months	0.4 (0.2-0.6)***	0.3 (0.2-0.5)***	-		-	
Meth use, injected or not, last 30 days *^1^	1.7 (1.0-3.0)**	1.8 (1.1-3.1)**	2.0 (1.1-3.5)**	1.9 (1.0-3.3)**	1.2 (0.2-7.0)	
Injected > 20 times/month	-		0.9 (0.6-1.5)		-	
Unsafe injection last 30 days	-		1.1 (0.6-1.8)		-	
Risky alcohol use*^2 ^	1.6 (1.0-2.4)**		1.3 (0.8-2.1)		3.2 (2.7-34.1)*	5.1 (0.9-30.1)*
Had sex when high on drugs or alcohol*^3 ^	2.0 (1.3-3.1)***	2.4 (1.5-3.8)***	1.8 (1.1-3.0)**	1.8 (1.1-3.0)**	9.5 (2.7-34.1)***	10.7 (2.6-43.9)***

* p< = 0.2

** p < = 0.05

*** p < = 0.01

*^1 ^99% of methamphetamine use was in the form of injection among IDUs

*^2 ^determined by an at-risk AUDIT-C score

*^3 ^had sex while participant or partner or both were drunk or high

*^4 ^also controlled for education, employment, homelessness, incarceration, living with family and having legal income. These variables were not significant

There was one significant interaction between drug injection and having sex when high on drugs or alcohol (p = 0.015). Having sex when high on drugs or alcohol was significantly associated with having multiple sex partners for both IDUs and non-IDUs. However, the magnitude of the relationship between having sex when high on drugs or alcohol and having multiple sex partners was greater for non-IDUs compared to IDUs (aOR = 10.7 vs. 1.8, respectively). No other interactions were identified.

### Correlates for Having Unprotected Sex

In multivariate logistic regression using all eligible participants, unprotected sex in the last six months was independently associated with being married, being a non-IDU, methamphetamine use and having sex when high on drugs or alcohol (Table 3). In analysis limited to IDUs, unprotected sex was associated with being married, using methamphetamine and having sex when high on drugs or alcohol. Among non-IDUs, unprotected sex was independently associated with risky alcohol use.

**Table 3:** Associations between substance use practices and unprotected sex for IDUs and non-IDUs

	All participants	N = 396	IDUs	n = 331	Non-IDUS	n = 65
	uOR	aOR*^4 ^	uOR	aOR*^4 ^	uOR	aOR*^4 ^
Male	0.8 (0.5-1.2)		1.0 (0.5-1.3)		1.1 (0.4-3.3)	
HIV	1.1 (0.7-1.8)		1.2 (0.7-2.0)		(insufficient n)	
Log age	1.5 (0.6-3.7)		1.3 (0.4-3.9)		4.6 (0.5-45.8)*	
Married/partnered	2.5 (1.6-3.9)***	2.5 (1.5-4.1)***	2.7 (1.7-4.5)***	2.4 (1.4-4.1)	1.9 (0.5-7.9)	
Any casual partner	0.9 (0.6-1.4)		0.8 (0.5-1.3)		0.7 (0.2-2.8)	
Injection drug use last 6 months	0.6 (0.3-1.1)**	0.4 (0.2-0.7)***	-		-	
Meth use, injected or not, last 30 days *^1^	1.9 (1.0-3.5)**	2.0 (1.1-3.9)**	1.9 (1.0-3.6)**	2.0 (1.0-3.9)***	2.8 (0.3-24.7)	
Injected > 20 times/month	-		1.0 (0.7-1.6)		-	
Unsafe injection last 30 days	-		2.1 (1.2-3.7)***		-	
Risky alcohol use*^2 ^	1.1 (0.7-1.8)		0.9 (0.5-1.4)		6.7 (1.4-31.4)**	6.7 (1.4-31.4)**
Had sex when high on drugs or alcohol*^3 ^	3.7 (2.3-5.8)***	3.8 (2.4-6.3)***	4.8 (2.8-8.1)***	4.6 (2.7-7.8)***	1.8 (0.6-5.5)	

* p< = 0.2

** p < = 0.05

*** p < = 0.01

*^1 ^99% of methamphetamine use was in the form of injection among IDUs

*^2 ^determined by an at-risk AUDIT-C score

*^3 ^had sex while participant or partner or both were drunk or high

*^4 ^also controlled for education, employment, homelessness, incarceration, living with family and having legal income. These variables were not significant

There was a statistically significant interaction between IDU and risky alcohol use (p = 0.009), in that non-IDUs who engaged in risky drinking had 6.7 greater odds of having unprotected sex than those who did not engage in risky drinking whereas there was no such relationship for IDUs. No other interactions were statistically significant.

### Correlates for Having Unprotected Sex within Sexual Partnerships Reported by Participants

In an analysis conducted at the partnership level, GEE was performed on 681 partnerships reported by the 396 IDUs and non-IDU participants. Having unprotected sex was independently associated with being married, not having a casual partner, methamphetamine use and having sex when high on drugs or alcohol (Table 4). When limited to the 541 partnerships reported by 331 IDUs, unprotected sex was independently associated with being married, not having a casual partner, methamphetamine use, unsafe injection and having sex when high on drugs or alcohol. In analysis of 140 sexual partnerships reported by 65 non-IDUs, unprotected sex was associated with older age.

**Table 4:** Associations between substance use practices and unprotected sex within all partnerships*^1 ^reported by IDUs and non-IDUs

	All participants = 396		IDUs = 331		Non-IDUS = 65	
	Partnerships = 681		Partnerships = 541		Partnerships = 140	
	uOR	aOR*^5 ^	uOR	aOR*^5 ^	uOR	aOR*^5 ^
Male	1.0 (0.7-1.5)		1.0 (0.6-1.5)		1.2 (0.5-2.9)	
HIV	1.1 (0.7-1.8)		1.2 (0.7-1.9)		3.0 (0.2-52.0)	
Log age	2.1 (0.9-4.8)*		1.5 (0.6-4.1)		6.1 (1.2-30.4)**	6.1 (1.2-30.4)**
Married/partnered	2.5 (1.7-3.7)***	1.9 (1.2-2.9)***	2.7 (1.7-4.2)***	1.9 (1.2-3.1)***	1.8 (0.7-4.9)	
Any casual partner	0.5 (0.4-0.7)***	0.5 (0.4-0.7)***	0.5 (0.3-0.7)***	0.5 (0.3-0.7)***	0.6 (0.3-1.1)*	
Injection drug use last 6 months	0.9 (0.5-1.4)		-		-	
Meth use, injected or not, last 30 days *^2^	1.7 (1.0-2.8)**	1.8 (1.1-3.2)**	1.7 (1.0-3.0)*	1.9 (1.0-3.5)**	1.6 (0.4-6.5)	
Injected > 20 times/month	-		1.0 (0.6-1.5)		-	
Unsafe injection last 30 days	-		2.0 (1.2-3.3)***	1.8 (1.0-3.2)**	-	
Risky alcohol use*^3 ^	1.0 (0.7-1.6)		0.9 (0.6-1.4)		3.0 (0.8-10.8)*	
Had sex when high on drugs or alcohol*^4 ^	2.4 (1.8-3.4)***	2.2 (1.6-3.1)***	3.1 (2.1-4.5)***	2.5 (1.7-3.7)***	1.2 (0.5-2.9)	

* p< = 0.2

** p < = 0.05

*** p < = 0.01

*^1 ^in GEE analysis limited to sexual partnerships reported by IDUs and non-IDUs

*^2 ^99% of methamphetamine use was in the form of injection among IDUs

*^3 ^determined by an at-risk AUDIT-C score

*^4 ^had sex while participant or partner or both were drunk or high

*^5 ^also controlled for education, employment, homelessness, incarceration, living with family and having legal income. These variables were not significant

## Discussion

Contrary to the initial hypothesis, IDUs were significantly less likely than non-IDUs who reported at least one IDU sex partner to have multiple sex partners and unprotected sex with casual partners. IDUs had fewer partners even after controlling for methamphetamine use, which was associated with higher number of sexual partners. This contrasts with findings from other studies that suggested that IDUs engaged in as much or more [9,30] sexual risk behaviors compared to individuals who did not inject.

IDUs were less likely than non-IDUs to engage in risky drinking, and risky drinking was not independently associated with sexual risk among IDUs. These results contrast with studies showing alcohol consumption to be greater among narcotic [31] or methamphetamine users [8,32] and to be associated with sexual risk taking among IDUs [33,34]. In a previous study, IDUs in Russia reported consuming lesser quantities of alcohol than IDUs in the United States [13]. Given that drug dependence and alcoholism have been observed to be co-occurring events in Russia [14] it is unclear whether the lesser prevalence of risky drinking among IDUs is due to a less risky alcohol consumption pattern or if it results from our failure to identify some other type of hazardous drinking that may occur in Russia [12]. It could be argued that the lower levels of risky alcohol use among IDUs could be due to underreporting as a result of social desirability bias or stigma and discrimination against IDUs. However, alcohol use is widely accepted in Russia and studies have shown that drinking is often used as a facilitator of sex and as a symbol of masculinity in the Russian culture [35]. In addition, there was no significant difference in the reported frequency of alcohol use between IDUs and non-IDUs in this study, it was only the pattern of risky drinking that significantly differed between the two groups. A possible explanation for these results is that alcohol is often used to remove pressure, relax and reduce stress whereas opiates are much more powerful relaxants than alcohol [35-39]. Since the majority of IDUs in this study injected heroin, they may have had a lesser need to resort to risky patterns of drinking in order to self-medicate [31] compared to non-IDUs. Another possible explanation for the lower levels of risky drinking among IDUs, comes from data showing that a majority 95% of IDUs in Russia are infected with hepatitis C virus (HCV) [40] and HCV infections often lead to liver diseases [41]. IDUs are also more likely to have HIV, to receive antiretroviral therapy or to have co-morbid conditions leading to poor health outcomes [42]. All of these factors could lead to a reduction of tolerance among IDUs for heavy patterns of drinking. The sedating effects of heroin and poor health conditions among IDUs could also account for the lower levels of sexual risk behaviors observed among IDUs compared to non-IDUs. In addition, the discrepancy between the two groups may be compounded by the increase in sexual risks that result from the greater risky alcohol use among non-IDUs [35].

Similar to previous findings, the present results show methamphetamine use to be the strongest predictor of having multiple sex partners and unprotected sex among this sample of heterosexual IDUs [6,8,43,44], while among non-IDUs, those sexual behaviors were associated with risky alcohol use [20,45].

The greater likelihood of reporting sex while high on drugs or alcohol among IDUs as well as among non-IDUs who had multiple sex partners probably reflects the greater amounts of substance use among IDUs and non-IDUs who had multiple sex partners. Only IDUs were more likely to forgo condom use when they had sex when high on drugs or alcohol. Similar to other studies of non-IDUs, the major risk factor associated with unprotected sex was the participant's general (i.e. monthly) pattern of risky drinking and not being drunk at the time of sex [46]. Thus, programs to increase condom use among IDUs need to address substance use patterns that occur in the context of sex, while among non-IDUs, general drinking patterns must be addressed [33].

No significant difference was observed in the sexual risk behaviors according to participants' gender, either overall or in the analysis stratified by drug use status suggesting greater similarities rather than differences between the sexual risks of men and women in this study. Given that previous studies have shown substance abusing women to be at greater risk for unsafe sex and STIs than substance abusing men [47-49] and studies in Russia have shown sex risk behaviors to be more likely among substance abusing men than among substance abusing women [35], it is also possible that subtle gender differences exist but were not identified in this study. Future research that takes into consideration motivations for risk behaviors and self efficacy for risk reduction might be able to provide further insight into these results.

The prevalence of STIs other than HIV has important epidemiologic implications given the propensity of STIs to increase the likelihood of HIV transmission [50,51]. Overall, STI prevalence among non-IDUs was comparable to the STI prevalence from the general population in St. Petersburg [52,53], and STI prevalence among IDUs in this study was similar to the prevalence found among IDUs in other countries [54,55]. The low STI prevalence might reflect a trend of reduced STIs being reported over the years in Russia [56-58]. The results have implications for behavior intervention research. The low STI prevalence may suggest that it may not be feasible or cost effective to use STIs outcomes in projects to reduce risk behaviors among these groups. However, given that the STI prevalence among participants is actually higher because other common STIs were not tested in this study, given the great prevalence of sexual risk behaviors among non-IDUs, and given that participants are part of vulnerable populations, efforts to detect and treat STIs among these groups need to continue.

This study had several limitations. Participants recruited into this study were IDUs and non-IDUs who reported at least one IDU sexual partner; results may not apply to non-IDUs who have never had sex with an IDU. Because the capacity of RDS to produce probability samples is limited [59] the generalizability of these results even within the sampled populations may also be limited. Sex risk behaviors may relate to substance use patterns; therefore, behaviors might have been different if the proportions of heroin, methamphetamine and alcohol users had been different. This study may have recruited individuals who were more comfortable participating in research projects rather than IDUs who were harder to reach. Conversely, certain or all risk behaviors may have been underreported due to social desirability or other types of bias. Last, due to questionnaire design, we could not investigate whether having sex when "high" was a specific effect of alcohol or of another drug, nor could we assess whether it was the participant, his or her partner or both who were drunk or high when sex occurred, nor could we assess the level at which participant was "high". Missing of data resulted in fewer observations in multivariate models which may increase risk of type II error; further, if missing data did not occur at random the potential for bias in ORs exists.

## Conclusions

Greater prevalence of sexual risk behaviors among non-IDUs who report partnerships with IDUs, compared to IDUs themselves, suggests a potential mechanism for spread of HIV beyond the IDU population. Interventions to reduce HIV transmission should address alcohol consumption among non-IDUs who report sexual partnerships with IDUs, and methamphetamine use and the propensity of having sex when high among IDUs.

## Competing interests

The authors declare that they have no competing interests.

## Authors' contributions

NA main contributor to conception, statistic analysis and drafting of this manuscript.

EW contributed to conception, statistic analysis and writing of this manuscript.

OT contributed to data acquisition, coordination and management, as well as quality control and interpretation of data.

TK contributed to data acquisition, quality control and interpretation and management of data.

SV carried out laboratory tests, interpretation and quality control of test results and contributed to management and interpretation of data.

AK contributed to conception and review of the manuscript.

RH contributed to conception, writing and review of the manuscript.

**All authors read and approved the final manuscript**.

## Pre-publication history

The pre-publication history for this paper can be accessed here:

http://www.biomedcentral.com/1471-2458/10/676/prepub
